# Improving Microalgal Biomass Productivity Using Weather-Forecast-Informed Operations

**DOI:** 10.3390/cells11091498

**Published:** 2022-04-29

**Authors:** Song Gao, Hongxiang Yan, Nathan Beirne, Mark Wigmosta, Michael Huesemann

**Affiliations:** 1 Marine and Coastal Research Laboratory, Pacific Northwest National Laboratory, Sequim, WA 98382, USA; nathan.beirne@pnnl.gov (N.B.); michael.huesemann@pnnl.gov (M.H.); 2 Energy and Environment Directorate, Pacific Northwest National Laboratory, Richland, WA 99354, USA; hongxiang.yan@pnnl.gov (H.Y.); mark.wigmosta@pnnl.gov (M.W.); 3 Department of Civil and Environmental Engineering, University of Washington, Seattle, WA 98104, USA

**Keywords:** microalgae, biomass productivity, dilution rate, weather-forecast-informed, photobioreactor

## Abstract

The operation of microalgal cultivation systems, such as culture dilution associated with harvests, affects biomass productivity. However, the constantly changing incident light and ambient temperature in the outdoor environment make it difficult to determine the operational parameters that result in optimal biomass growth. To address this problem, we present a pond operation optimization tool that predicts biomass growth based on future weather conditions to identify the optimal dilution rate that maximizes biomass productivity. The concept was tested by comparing the biomass productivities of three dilution scenarios: standard batch cultivation (no dilution), fixed-rate dilution (harvest 60% of the culture every three days), and weather-forecast-informed dilution. In the weather-forecast-informed case, the culture was diluted daily, and the dilution ratio was optimized by the operation optimization tool according to the future 24 h weather condition. The results show that the weather-forecast-informed dilution improved the biomass productivity by 47% over the standard batch cultivation and 20% over the fixed-rate dilution case. These results demonstrate that the pond operation optimization tool could help pond operators to make decisions that maximize biomass growth in the field under ever-changing weather conditions.

## 1. Introduction

The rising level of anthropogenic greenhouse gases in the atmosphere, mainly CO_2_, is of increasing concern worldwide. Irreversible damage to ecosystems is possible as global warming surpasses 1.5 °C if no effective mitigation is achieved in the coming decades [1]. Biofuels can contribute to a solution by providing carbon-neutral energy for growing consumer societies. Microalgae, due to their unique advantages in biomass production, such as high growth rates, their ability to grow on non-arable land and non-potable water, and their capability to remove waste nutrients from water and carbon from the atmosphere, are regarded as one of the most promising feedstocks for conversion to biofuels [2,3].

However, according to a recent techno-economic analysis, the current low biomass productivity is the major barrier to achieving economically viable microalgal biofuels [4]. Although substantial progress has been made over the years in understanding the relationship between microalgal growth and environmental factors [5,6], using them to guide cultivation in outdoor systems such as raceway ponds has been difficult. The main challenge lies in the complex interaction among the biological property of the cultivated strain, the culture status (e.g., biomass concentration, culture depth), the varying environmental conditions (e.g., light, temperature), and the impact of pond operation (e.g., dilution). Currently, pond operation decisions are usually made following a prefixed routine or based on operators’ experience. It is difficult to verify whether the operation is optimal or not for biomass growth without conducting resource-intensive and time-consuming experiments. Even if such experiments are completed, the lessons learned may not be applied directly to cultivations at a different location or in a different season because weather conditions are different. Although, as demonstrated by De-Luca et al. [7,8,9], theocratical modeling results of biomass growth indicated that optimizing pond operation according to weather change could significantly improve biomass productivity, weather-responsive operations are not commonly applied in current microalgal cultivations due to the abovementioned difficulties.

Recently, we proposed a biomass modeling system for microalgae cultivation in outdoor raceway ponds that consists of two components: a weather condition module and a biomass growth module [10]. The weather condition module predicts incident light intensity and water temperature based on the data collected by weather stations. These outputs are fed to the biomass growth module to predict biomass growth. The biomass growth model adopted in the biomass growth module was built upon experimentally determined strain-specific parameters. Its accuracy was validated by raceway pond cultivation data [11]. In theory, the system can predict future biomass productivity as a function of operational parameters such as culture dilution and culture depth by using forecasted weather conditions and can be a tool for the operators to refer to before making operational decisions. Therefore, the goal of this study is to validate this concept. Once validated, the proposed biomass modeling system can be incorporated into the biomass assessment tool, a framework that can be used to provide a national assessment of microalgal cultivation strategies by accounting for algal strain selection, climate conditions, saline and wastewater resources, CO_2_ resources, land prices, and many other factors [12,13,14].

In pond cultures, light attenuates exponentially with depth, and a light-limited zone can develop and occupy a large portion of the culture [15]. Culture dilution, by removing a fraction of the biomass and replacing the culture with fresh medium, lowers biomass concentration and allows deeper light penetration, hence it reduces light-limited growth and increases biomass productivity. Previous studies have demonstrated that the dilution rate (percent and frequency of biomass removal) is an important operational parameter that has a significant impact on biomass productivity. For instance, a daily dilution ratio of 30% could improve biomass productivity by over 46% with respect to batch cultivation [16], and an optimal dilution rate could more than double biomass productivity [17]. However, these and many other similar studies were conducted under well-controlled indoor conditions where light and/or temperature were held constant. The conclusions are not transferrable to outdoor cultivation, which is subjected to uncontrolled and constantly changing environmental conditions. The relationship between dilution rate and biomass productivity can change frequently and drastically as the weather varies from day to day.

In this study, the aforementioned modeling system was modified and used as a weather-forecast-informed pond operation optimization tool. Three operational scenarios, standard batch cultivation, fixed-rate dilution, and weather-forecast-informed dilution, were applied to microalgal cultures grown in a climate simulation photobioreactor that replicates the outdoor pond conditions. Biomass productivities of the three operational scenarios were compared to validate the usefulness of the operation optimization tool.

## 2. Materials and Methods

### 2.1. Strain

The green alga *Chlorella sorokiniana* DOE1412, a strain also known as *Chlorella sorokiniana* UTEX B 3016, was cultivated in this study. The strain was obtained from the National Alliance for Advanced Biofuels and Bioproducts (NAABB) cultivation team [18,19].

### 2.2. Outdoor Pond Condition Simulation

A bench-scale photobioreactor, the Laboratory Environmental Algae Pond Simulator (LEAPS, [20]), was employed in the present study, which accurately replicated light and temperature conditions in outdoor raceway ponds for biomass growth. There are six identical columns in the LEAPS, allowing duplicate cultures for each of the three operational scenarios to be grown side by side. Each LEAPS column holds a 1 L culture at 20 cm depth. All the cultures were grown in BG-11 medium [21], and the pH was maintained at 7–7.5 by continuous sparging with 0.8–1.2% CO_2_-enriched air. The entire system was surrounded by a thick black curtain to block external light, and each column was wrapped with black vinyl paper to ensure that the only light source was the light panel on the top.

**Figure 1:**
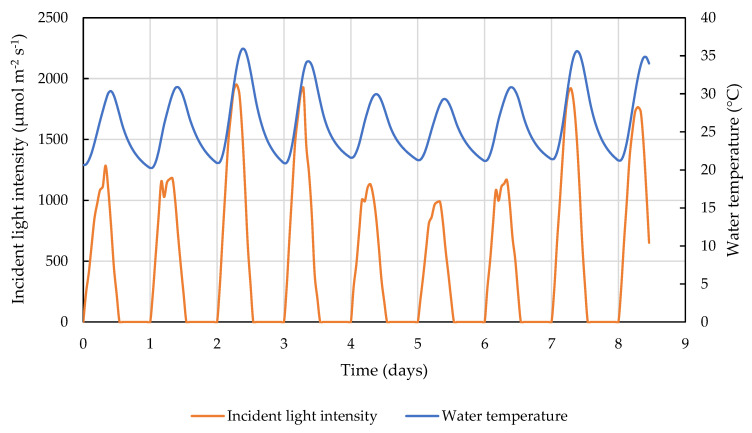
Incident light intensity and water temperature scripts used for growth simulation in the LEAPS photobioreactors. Time 0 corresponds to 25 August 2013 6:00 am.

The incident light and temperature conditions for the growth simulation in the LEAPS are shown in Figure 1. The diurnally fluctuating incident light intensity was obtained from the Phase 2 of the North American Land Data Assimilation System (NLDAS2, [22]) between 25 August 2013 and 1 September 2013 near Mesa, Arizona. The water temperature was simulated for a 20 cm deep outdoor pond culture by the Modular Aquatic Simulation System in Two Dimensions (MASS2, [23]) driven by the NLDAS2 meteorological data, including solar radiation, air temperature, wind speed, and dew point temperature.

### 2.3. Operational Scenarios

#### 2.3.1. Baseline Scenario

The culture was grown in standard batch culture mode. No dilution was applied, and biomass was harvested at the end.

#### 2.3.2. Fixed-Rate Dilution Scenario

The cultures were diluted by removing 60% of the culture every 3 days and replenishing with fresh medium. This type of operation is commonly adopted in microalgal cultivation.

#### 2.3.3. Weather-Forecast-Informed Dilution Scenario

Every day at 18:00, biomass growth in the next 24 h was simulated as a function of dilution ratio by the pond operation optimization tool using the weather conditions in the next 24 h. The dilution ratio that resulted in the highest future biomass productivity was selected and applied to the cultures. Because the incident light, water temperature, and biomass concentration changed from day to day, the optimal dilution ratio was adjusted accordingly.

### 2.4. Data Collection and Processing

Every day at 18:00, samples were removed from each LEAPS column after adding distilled water to compensate for evaporative losses. If no dilution occurred, 25 mL samples were used for biomass concentration measurements. If dilution occurred, additional culture was removed to match the dilution ratio at either 60% for the fixed-rate dilution case or the optimal ratio determined by the pond optimization tool. Fresh medium was then added to bring the culture depth back to 20 cm. After one week, the experiment was repeated once under the same growth and operational conditions.

Culture biomass concentration was measured as ash-free dry weight (AFDW, mg L^−1^). A 20 mL sample was filtered onto a pre-combusted and pre-weighed Whatman GF/F microfiber filter. The filter with biomass was dried at 105 °C overnight and combusted at 540 °C for at least 2 h. The mass change following combustion divided by the sample volume (20 mL) represented the combustible organic matter, reported as AFDW.

The areal biomass productivities for the three scenarios were calculated by multiplying the slope of the total biomass versus time curve (from the first point to the last point) by the culture depth (20 cm). For the baseline case, no biomass was harvested, therefore, the total biomass was equal to the biomass in the reactor. For the other two cases, the total biomass was the sum of biomass in the reactor and the harvested biomass:

(1) $$BT_{n}=C_{n}\times V+\sum_{i=0}^{n}BH_{i}$$

where BT_n_ is the total biomass on the nth day (mg), C_n_ is the biomass concentration in the reactor (mg L^−1^) on the nth day, V is the volume of the culture (1 L), and $\sum_{i=0}^{n}{BH}_{i}$ is the sum of the biomass harvested from day 0 to the nth day (mg).

Average light intensity during the light period was estimated by the biomass growth module for each day based on the light attenuation algorithm in the model (Equation (2)). This variable measures the overall underwater light condition.

(2) $$I_{\mathrm{average}}=\frac{1}{t}\int_{0}^{t}\frac{1}{d}\int_{0}^{d}I_{t}{(z)d}_{z}d_{t}$$

where t represents the light period from sunrise to sunset each day, *d* is the culture depth (20 cm), and $I_{t}\left(z\right)$ is the light intensity at depth z at time t (μmol m^−2^ s^−1^), which is determined by Equation (3).

(3) $$I_{t}{(z)=I}_{0}{(t)e}^{{-k}_{a}\cdot C(t)\cdot z}$$

where $I_{0}\left(t\right)$ is the incident light intensity at time t (μmol m^−2^ s^−1^), k_a_ is the strain-specific light extinction constant (mg^−1^ m^−1^), and C(t) is biomass concentration at time t (mg L^−1^).

## 3. Results and Discussion

**Figure 2:**
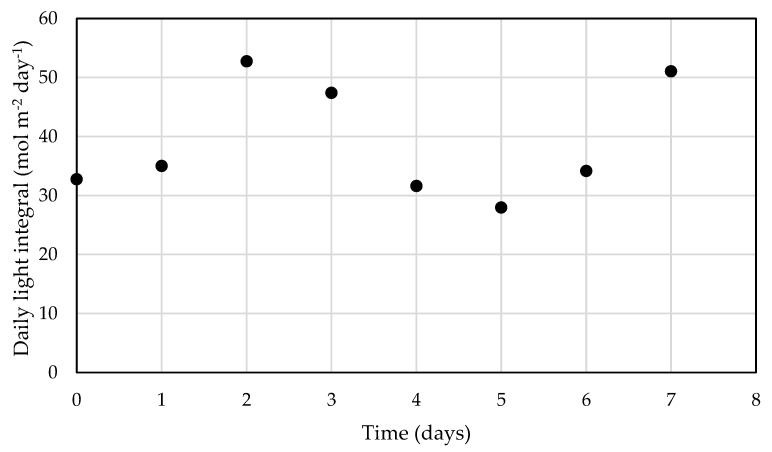
Daily light integral (DLI) as a function of time for the light script used in the LEAPS climate simulation cultivation.

Before introducing the results, it should be noted that, for this proof-of-concept study conducted in the indoor photobioreactor, the incident light and water temperature scripts were used to replace the outputs of the weather forecasting module, equivalent to a perfect forecast of these parameters. To better present the variation in light availability from day to day over the course of the experiment, daily light integral (DLI) is plotted as a function of time (Figure 2). Days 2, 3, and 7 were sunny days, and the DLI on those days was near 50 mol m^−2^ day^−1^. The rest of the week was cloudy, and DLI was significantly lower, varying between 28 and 35 mol m^−2^ day^−1^. Although Arizona is one of the sunniest states in the United States, variations such as these are not uncommon and can be even more drastic in the monsoon season from June to September.

**Figure 3:**
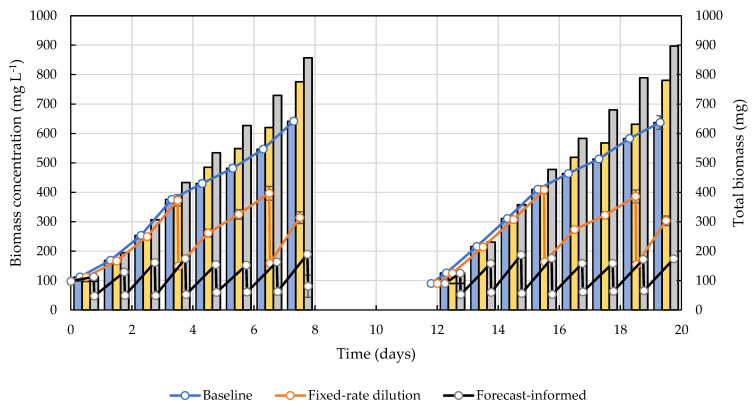
The biomass growth curves for the three dilution cases. The solid lines represent the biomass concentration in the LEAPS bioreactor as a function of time. The sharp decreases in AFDW in the fixed-dilution and forecast-informed dilution cases were due to dilution by biomass harvesting. The bars represent the total biomass (the sum of the biomass that remained in the reactor and harvested) as a function of time. Blue for the baseline case, orange for fixed-dilution case, and gray for the forecast-informed case. Error bars represent the standard error of the mean of the duplicated cultures.

The biomass growth curves of the two repeated batch culture runs are presented in Figure 3. The solid lines represent the biomass measured in the bioreactor over time. The bars represent the total biomass, equal to the sum of the biomass in the reactor and the biomass harvested. For the baseline culture, because there was no harvest, the total biomass (blue bar) was equal to the biomass in the bioreactor (blue dots). For the fixed-rate dilution scenario, the biomass concentration of the culture (orange line) decreased twice due to two 60% dilutions, i.e., on Day 3 and Day 6 for the first batch run and on Day 15 and Day 18 for the second batch run. The total biomass (orange bar) surpassed the baseline after the first harvest and remained the second highest among the three operational scenarios. For the weather-forecast-informed dilution scenario, the culture was harvested every 24 h, and the dilution ratios were 58%, 62%, 80%, 70%, 61%, 60%, 62%, and 71% over the eight days, respectively, as determined by the operation optimization tool. Although the biomass concentration in the reactor remained the lowest, the accumulated total biomass, shown by the gray bar, achieved the highest value at the end.

The average biomass productivities of the duplicate runs were 14.8 ± 0.3, 18.2 ± 0.5, and 21.7 ± 0.3 g m^−2^ day^−1^ for the baseline, fixed-dilution, and weather-informed dilution cases, respectively. The weather-forecast-informed optimization led to a 47% increase in biomass productivity over the baseline scenario and a 20% increase over the fixed-rate dilution scenario. This result demonstrates that the proposed pond operation optimization tool adjusted the dilution rate dynamically based on future weather conditions and was able to improve biomass productivity substantially, thereby possibly reducing biofuels costs.

**Figure 4:**
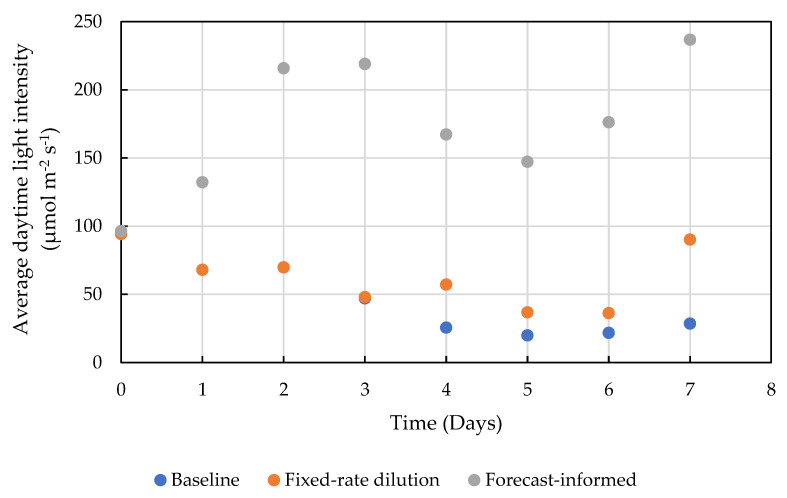
Average light intensity in the culture each day for the three operation cases according to the biomass growth model. The average light intensities in the first 3 days of the baseline case overlapped with those of the fixed-dilution case, and thus are not visible.

The improvement in biomass productivity was primarily attributed by the increased average light intensity as the culture was being diluted. As shown in Figure 4, the weather-forecast-informed dilution operation kept the average light intensity significantly higher than the other two operations. During cloudy days (Day 4 and 5), the operation optimization tool suggested high dilution ratios (80% and 70%) to minimize the impact of less light, which kept the average light intensity above 150 μmol m^−2^ day^−1^, 365% and 550% higher than that in the baseline scenario, and 356% and 192% higher than that in the fixed-rate dilution scenario.

**Figure 5:**
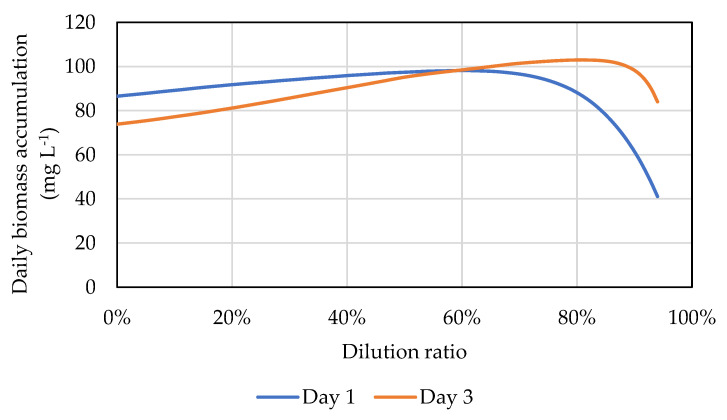
The predicted daily biomass growth as a function of the dilution ratio predicted by the operation optimization tool. The dilution ratio that yields the most biomass growth is 58% for Day 1 and 80% for Day 3.

However, it should be pointed out that a high dilution ratio does not necessarily lead to high biomass productivity. Increasing the dilution ratio above the optimal level lowers biomass productivity. In an over-diluted culture, a large portion of the culture may be exposed to over-saturated light intensity, which may cause photoinhibition and slow down biomass growth [24]. Moreover, if the culture is over-diluted, biomass productivity is limited because the number of cells that are able to divide is low. Figure 5 shows an example of how the predicted daily biomass growth (the biomass accumulated in 24 h after dilution) changes with the dilution ratio on Day 1 and Day 3. The optimal dilution ratio for Day 1 is 58%. Due to the coming cloudy day on Day 4, the optimal dilution ratio on Day 3 increased to 80%. On both days, the biomass productivity increases with increasing dilution ratio below the optimal level due to improved light penetration, but a further increase in dilution ratio above the optimal level causes a decline in biomass productivity.

Although the weather-forecast-informed pond optimization tool improved biomass productivity significantly relative to the baseline and fixed-rate dilution scenarios, we are aware of the potential negative economic implications associated with the removal of larger harvest volumes from the culture. Although culture medium can be recycled, the energy required for moving the water and harvesting the biomass is likely higher than the baseline case. As a proof-of-concept study, the primary focus of the present work was on improving biomass productivity, thus, a technoeconomic analysis (TEA) was not included. In future work, a TEA will be incorporated into the optimization tool, and optimal operation, e.g., dilution ratio and frequency and other operation parameters, can be determined under economic constraints in an outdoor setting using real-time weather-forecast data.

In conclusion, the weather-forecast-informed pond operation optimization tool showed great potential for improving biomass cultivation in an outdoor environment. By solely implementing weather responsive optimization of culture dilution, an improvement of 47% in biomass productivity relative to standard batch cultivation was achieved.

## Data Availability

The incident light intensity, water temperature, and biomass growth data are available upon request.
